# Prevalencia de osteoesclerosis idiopática de pacientes atendidos en un centro radiológico de Ayacucho entre los años 2016 y 2018

**DOI:** 10.21142/2523-2754-0903-2021-071

**Published:** 2021-10-06

**Authors:** Paul Marcelo Ñahuincopa López

**Affiliations:** 1 Carrera de Odontología, Universidad Científica del Sur. Lima, Perú. marelo53@hotmail.com Universidad Científica del Sur Carrera de Odontología Universidad Científica del Sur Lima Peru marelo53@hotmail.com

**Keywords:** osteoesclerosis, radiografía panorámica, tomografía computarizada de haz cónico, mandíbula, osteoesclerosis, panoramic radiography, cone beam computed tomography, mandible

## Abstract

**Objetivo:**

El objetivo de este trabajo fue determinar, describir e identificar la prevalencia de las imágenes radiopacas compatibles con osteoesclerosis idiopática, evaluada con radiografías panorámicas digitales tomadas en pacientes de 18 a 50 años que acudieron entre los años 2016 y 2018 a un centro radiológico privado en la ciudad de Huamanga (Ayacucho, Perú).

**Materiales y métodos:**

El diseño fue descriptivo, transversal y retrospectivo. La muestra estuvo conformada por 500 radiografías panorámicas digitales tomadas entre el 1 de enero del 2016 y el 31 de diciembre del 2018, correspondientes a la población objetivo, de un grupo etario entre 18 y 50 años de edad, natural de la región de Ayacucho, Perú, que acudieron al Centro Radiológico. Las radiografías obtenidas fueron observadas y analizadas a través del programa Romexis viewer versión 5.3, y se anotaron los resultados en una ficha de recolección de datos. La prueba de chi-cuadrado fue utilizada para establecer la asociación entre las variables evaluadas, p < 0,05.

**Resultados:**

De la muestra total de 500 radiografías panorámicas digitales analizadas la prevalencia de osteoesclersis idiopática fue del 17.4%, de las cuales el 12% corresponden al sexo femenino y el 5.4% al sexo masculino. De acuerdo a la edad, la presencia de osteoesclerosis idiopática fue más prevalente en la segunda década de vida. **C**

**onclusiones:**

Es importante tener un criterio diagnóstico diferencial claro al distinguir las diferentes radiopacidades como la OI, que se pueden presentar en los maxilares, haciendo un registro preciso de sus características morfométricas y seguimiento en el tiempo, teniendo en cuenta su existencia y sus implicancias en los tratamientos dentales a futuro.

## Abstract (en)

**Objective:**

The objective of this study was to determine, describe and identify the prevalence of radiopaque images compatible with idiopathic osteosclerosis in digital panoramic radiographs taken in patients between the ages of 18 and 50.

**Materials and methods:**

The study design was descriptive, cross-sectional and retrospective. The sample consisted of 500 digital panoramic radiographs taken between January 1, 2016 and December 31, 2018, in the target population aged between 18 and 50 years from Ayacucho - Peru, attended at a private radiological center. The radiographs obtained were analyzed using the Romexis viewer version 5.3 program, and the results were recorded on a data collection sheet. The Chi-square test was used to establish associations among the variables evaluated. A P <0.05 was considered significant.

**Results:**

500 digital panoramic radiographs were analyzed, showing a prevalence of idiopathic osteosclerosis of 17.4%, of which 12% were female and 5.4% male, and according to age, the presence of idiopathic osteosclerosis was more prevalent in the second decade of life.

**Conclusions:**

It is important to have a clear differential diagnostic criterion when distinguishing the different radiopacities such as idiopathic osteosclerosis, which can occur in the jaws, making a precise record of the morphometric characteristics and monitoring over time, taking into account their existence and implication in dental treatments.

## INTRODUCCIÓN

Las imágenes radiopacas que se presentan en radiología oral y maxilofacial son de etiología multifactorial. Dentro de este grupo de imágenes conocidas como condensaciones óseas (CO) se encuentra la osteoesclerosis idiopática (OI), inicialmente reportada por Stieda en 1905 y ratificada por Fischer en 1912 ^1^, definida desde sus inicios como un área focal ^2^. Es importante el diagnóstico diferencial para no confundirla con otras radiopacidades óseas presentes en los maxilares, sobre todo en la mandíbula ^3^^-^^8^, aunque puede presentarse también en diferentes partes del esqueleto humano, siendo más frecuente en los huesos largos y los huesos de la cadera ^3^^-^^8^. Es más común hallarla entre la segunda y la cuarta década de vida; posteriormente es menos frecuente, pero también puede presentarse ^8^^,^^9^.

Estas imágenes son de fondo homogéneo radiopaco, bordes regulares, sin halo o cortical, de tamaño variable, únicos o múltiples, de etiología en algunos casos desconocida ^10^, de la cual deriva parte de su nombre ^11^, aunque algunos investigadores relacionan su origen con procesos inflamatorios presentes en tratamientos de ortodoncia, estrés oclusal o como parte del desarrollo óseo ^4^. No se encuentra relacionada con alteraciones como displasias, neoplasias, trastornos de orden sistémico o secuelas de fracturas ^12^^-^^14^. Un diagnóstico diferencial certero determinaría con precisión la prevalencia de la OI.

Dentro de los estudios en la región sudamericana, en enero del 2020, los investigadores Pflucker-Ballón *et al*. ^12^, de una muestra de 1500 radiografías panorámicas, obtenidas de una población de Lima-Perú, reportaron una prevalencia del 8,5% de OI, lo que coincide en la descripción de las características radiográficas con lo ya reportado en la literatura especializada. En el 2017, Fuentes *et al*. ^13^, en una muestra compuesta por 1000 radiografías panorámicas obtenidas de una población chilena, reportaron una prevalencia del 2,7% de aparición de la OI, y concluyeron que los datos obtenidos se corroboraron con estudios previos. En el 2018, en México, Ledesma-Montes *et al*. ^14^, de un total de 6340 radiografías analizadas, se reportó una prevalencia del 5,6% de OI, y coincide en sus características morfométricas con otros estudios reportados.

Son contados aún los estudios derivados de la casuística, acerca de la prevalencia de OI, y existen pocos estudios regionales similares en el Perú. Según los antecedentes, la OI se reportó con mayor frecuencia en poblaciones de origen africano, japoneses, chino e indochinos ^15^. Es importante tomar en cuenta estos datos para el diagnóstico en países multiétnicos.

De acuerdo con lo expuesto, cabe tener en cuenta que existe una alta diversidad genética en la población peruana, por lo que resulta necesario determinar la prevalencia de la OI a través del estudio de las radiografías panorámicas digitales en una población mestiza de adultos de la región Ayacucho, que presentan un alto componente indígena andino.

## MATERIALES Y MÉTODOS

El diseño de estudio fue descriptivo, transversal y retrospectivo. La muestra estuvo conformada por 500 radiografías panorámicas digitales, tomadas entre el 1 de enero del 2016 y el 31 de diciembre del 2018, correspondientes a la población objetivo, de un grupo etario entre 18 y 50 años de edad, natural de la región de Ayacucho (Perú), que acudieron al centro radiológico Corazón de Jesús.

Se evaluó la presencia de osteoesclerosis idiopática, se registró el sexo y la edad, y se recolectaron esos datos en una ficha. Así mismo, previamente, el investigador fue calibrado/capacitado por un especialista en radiología oral y maxilofacial; este proceso fue realizada en 100 radiografías panorámicas digitales y el valor de Kappa interobservador fue de 0,9 y el intraobservador, de 1.

Las radiografías que se obtuvieron en la muestra fueron tomadas con un equipo radiográfico digital de procedencia coreana, de la marca Point Nix, modelo Point 800S HD Plus, tomadas con un miliamperaje de 10 mA y un kilovoltaje de 70 kV, durante un tiempo aproximado de 17 segundos para la toma de la RPD. El equipo contaba con un sensor super OLED que brinda imágenes de mejor resolución con menos del 1% de distorsión.

**Figura 1:**
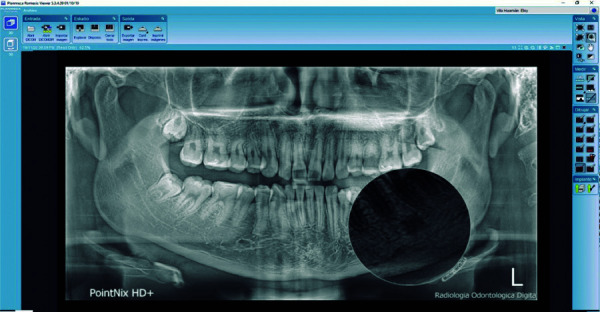
Imagen compatible con osteoesclerosis idiopática de bordes regulares y fondo homogéneo.

**Figura 2:**
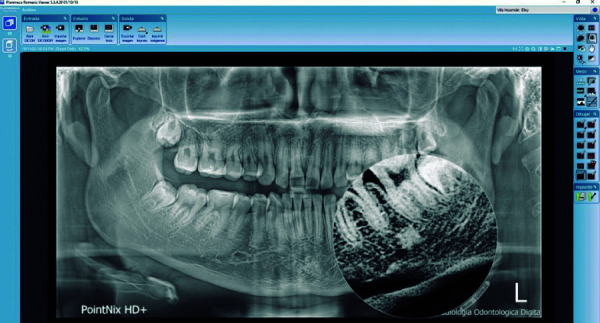
Imagen compatible con osteoesclerosis idiopática vista en una radiografía con colores invertidos para poder evidenciar con más claridad sus bordes regulares y fondo homogéneo

**Figura 3:**
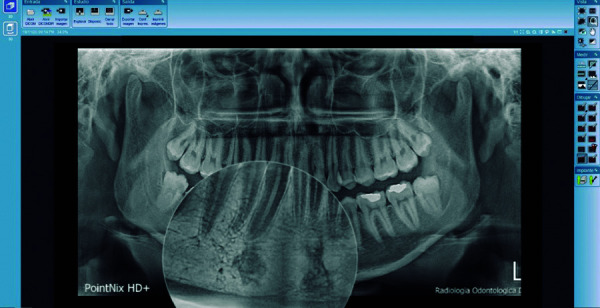
Imagen compatible con osteoesclerosis idiopática adyacente al foramen mentoniano, bordes irregulares y fondo homogéneo.

Las imágenes obtenidas fueron procesadas con el programa informático para el procesamiento de las imágenes CDX View DICOM, proporcionado con el equipo Point 800S HD Plus, y valoradas en un monitor de la marca Asus, modelo MX279H, tecnología Sonic Master, de 27 pulgadas, full HD AH-IPS sin marco, con 178° de ángulo de visión. Se analizó las RPD bajo una luz adecuada para su interpretación y lectura, con el visualizador Romexis (Planmeca), versión 5.3 (Figuras 1-3).

Los datos obtenidos del presente proyecto de investiga-ción fueron procesados a través del programa estadístico para el análisis de las ciencias sociales IBM SPSS Statistics (Statistical Package for the Social Sciences), versión 25, en castellano. Se plantearon las tablas de frecuencias, los porcentajes en la estadística descriptiva y la prueba de chi-cuadrado en la estadística analítica para establecer la asociación entre las variables evaluadas. Se asumió un nivel de significancia de p < 0,05.

## RESULTADOS

**Figura 4:**
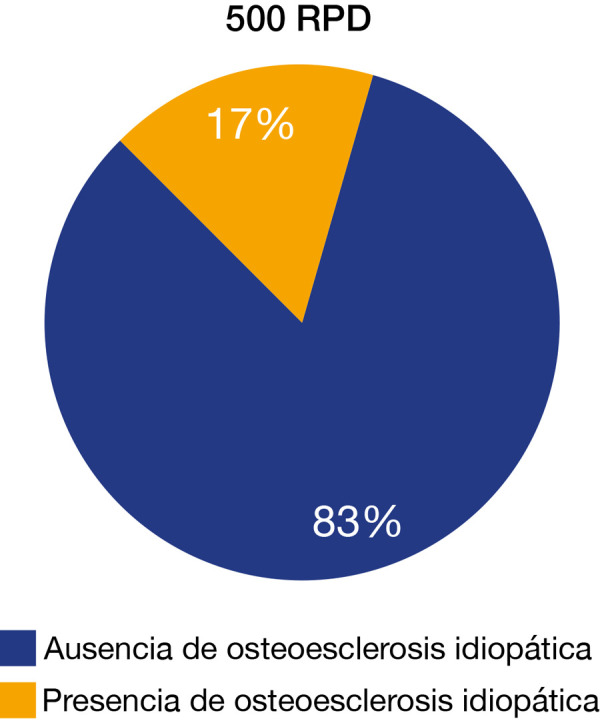
Prevalencia de osteoesclerosis idiopática

En la Tabla 1 y la Figura 4, se analizó una muestra total de 500 RPD, presentando una prevalencia de OI correspondiente a un total de 87 RPD, equivalente a un 17,4% del total de la muestra, con 67 RPD con presencia única de OI y 20 RPD con presencia múltiple de OI.

**Tabla 1:** Prevalencia de la osteoesclerosis idiopática

*N (100%)							
Osteoesclerosis idiopática	500	Ausencia		413 (82,6%)	
		Presencia	87 (17,4%)	Única 67	Múltiple 20

*N: tamaño de la muestra

**Figura 5:**
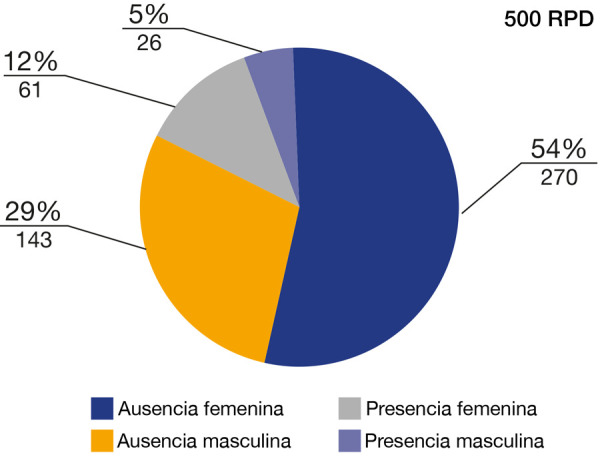
Prevalencia de osteoesclerosis idiopática con respecto al sexo

En la Tabla 2 y la Figura 5, de la muestra total de 500 RPD analizadas, 331 RPD correspondían al sexo femenino, 270 RPD con ausencia de OI y 61 RPD con presencia de OI, mientras que 169 RPD correspondían al sexo masculino, 143 RPD con ausencia de OI y 26 RPD con presencia de OI.

**Tabla 2:** Prevalencia de osteoesclerosis idiopática con respecto al sexo

	*N (100%)		Sexo		**Valor “p”
			Femenino	Masculino		
Osteoesclerosis idiopática	413 (83%)	Ausencia	270 (54%)	143 (29%)	0,721
	87 (17%)	Presencia	61 (12%)	26 (5%)	
Total	500 (100%)		331 (66%)	169 (34%)	

*N: tamaño de la muestra. **Prueba de chi-cuadrado de Pearson

**Figura 6:**
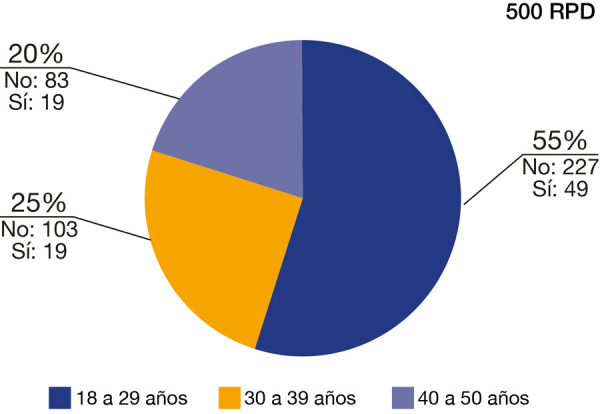
Prevalencia de osteoesclerosis idiopática con respecto a la edad

En la Tabla 3 y la Figura 6, del total de la muestra de 500 RPD, 276 RPD corresponden al grupo etáreo n.^o^ 1, de 18 a 29 años, con 227 RPD con ausencia de OI y 49 RPD con presencia de OI; el grupo etáreo n.^o^ 2, de 30 a 39 años, con 103 RPD con ausencia de OI y 19 RPD con presencia de OI; el grupo etáreo n.^o^ 3, de 40 a 50 años, con 83 RPD con ausencia de OI y 19 RPD con presencia de OI.

**Tabla 3:** Prevalencia de osteoesclerosis idiopática con respecto a la edad

	*N (100%)		Edad			**Valor “p”
			18 a 29 años	30 a 39 años	40 a 50 años	
Osteoesclerosis idiopática	413 (82,6%)	Ausencia	227 (54,9%)	103 (24,9%)	83 (20,1%)	0,414
	87 (17,4)	Presencia	49 (56,3%)	19 (21,8%)	19 (21,8%)	
Total	500 (100%)		276 (55,2%)	122 (24,4%)	102 (20,4%)	

*N: tamaño de la muestra, **Prueba de Chi-cuadrado de Pearson

## DISCUSIÓN

La presencia de la OI fue reportada por primera vez en los estudios de Stieda ^1^, en 1905, y ratificado por las observaciones de Fisher ^1^, en 1912. Su hallazgo se produjo de forma accidental ^31^^,^^34^, durante la observación de la RPD ^3^^,^^19^^,^^22^. Algunos investigadores consideran a la OI como una variante anatómica del desarrollo óseo normal, durante el proceso de desarrollo y maduración. En un gran porcentaje de casos es asintomática y, por lo general, no requiere tratamiento, solo seguimiento radiográfico a lo largo del tiempo ^2^^,^^12^^-^^14^^,^^30^^,^^31^^,^^34^.

**Figura 7:**
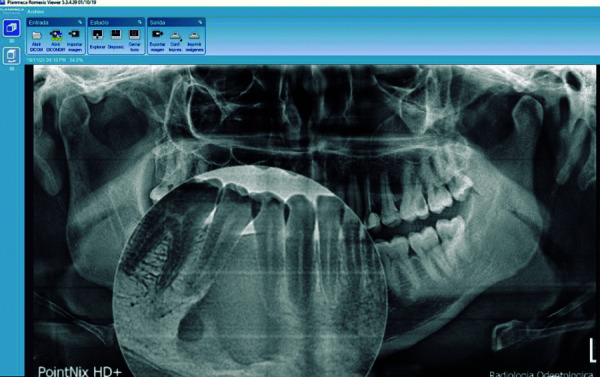
Osteoesclerosis idiopática de tamaño considerable que se extiende desde la cresta alveolar hasta el foramen mandibular.

La literatura científica a nivel internacional reporta diversos porcentajes de prevalencia de OI en radiografías panorámicas, como 1,96% ^25^, 2,44% ^19^, 2,7% ^26^, 2,8% ^13^, 2,84% ^27^, 4,7% ^28^, 5,4% ^29^, 5,6% ^30^, 6% ^31^, 7,5% ^21^, 9,5% ^(6, 32)^, 11,3% ^33^ y 11,8% ^7^. Las medidas referenciales promedio con respecto al área de la OI son de 33,9 ± 20,1 mm2, con una altura de 7,7 ± 3,1 mm y un ancho de 6,6 ± 3,1 mm; siendo la distancia desde la OI hasta la línea media mandibular de 26,6 ± 10,7 mm y 9,7 ± 3,7 mm al borde mandibular ^13^ (Figura 7). 

A nivel nacional, se reportó, en enero del 2020, el estudio realizado por los investigadores Pflucker-Ballón y Fiori-Chíncaro ^12^, quienes analizaron una muestra total de 1500 RPD, obtenidas de una población de la capital peruana en el distrito de Miraflores, y que reportaron una prevalencia de OI en 127 RPD que corresponde a un porcentaje del 8,5% del total de la muestra analizada, con presencia única ^12^. A nivel regional no existen estudios acerca de la prevalencia de OI.

Los resultados reportados por la literatura a nivel mundial sobre la prevalencia de la OI con respecto al sexo determinaron que tiene predilección por el sexo femenino con respecto al sexo masculino ^9^^,^^12^^,^^13^^,^^19^^,^^28^.

La prevalencia de la OI con respecto a la edad muestra que es más prevalente entre la segunda y la cuarta década de vida ^9^^,^^12^^,^^13^; sin embargo, también está presente durante la quinta década de vida en adelante ^13^^,^^30^.

La presente investigación analizó una muestra total de 500 RPD, tomadas en la ciudad de Huamanga, región Ayacucho, en el centro radiológico Corazón de Jesús, entre los años 2016 y 2018, se encontró la presencia de OI en 87 RPD, con una prevalencia del 17,4% con respecto al total de 500 RPD. De las 87 RPD señaladas, 67 RPD (77%) reportaron presentación única de OI y 20 RPD (23%), presencia múltiple de OI. Si bien es cierto que se trata de un porcentaje alto con respecto a lo reportado en la literatura para una población peruana, hay factores por considerar que pueden incidir en una alta prevalencia, como la alta diversidad genética en la población peruana, en general. En particular, al analizar una población mestiza de adultos de la región Ayacucho, el estudio de Guio *et al*. ^35^ halló en las capitales de departamento como Huamanga que la población mestiza presenta un alto componente indígena andino concentrado, que deriva de la poblaciones indígenas cercanas. Este fenómeno se da en Ayacucho y Cusco, a diferencia de la ciudad de Lima, donde se realizó un estudio de prevalencia de OI por parte de Pflucker-Ballón *et al*. ^12^, en el distrito de Miraflores, y se encontró que el componente indígena tiene un origen mixto derivado de las diferentes regiones del Perú y, adicionalmente, del extranjero, por ser un distrito eminentemente turístico. Esto concuerda con lo reportado acerca de la variabilidad multiétnica reportada en estudios de otras latitudes ^5^.

La prevalencia con respecto al sexo reportada en esta investigación evidenció predilección por el sexo femenino en una proporción de dos a uno con respecto al sexo masculino, lo que coincide con los resultados reportados por la literatura científica internacional y nacional ^9^^,^^12^^,^^13^^,^^17^^,^^19^^,^^20^^,^^36^, pero difieren de los estudios reportados por Zayet *et al*. ^37^ y Demir *et al*. ^38^, que reportaron una distribución equitativa con respecto al sexo en la prevalencia de la OI ^37^^,^^38^.

Los resultados reportados por esta investigación, respecto de la prevalencia de la OI relacionada con la edad, concuerdan con los rangos etarios reportados por investigaciones internacionales y nacionales, que refieren una mayor prevalencia entre la segunda y la cuarta década de vida (en esta investigación se halló una mayor prevalencia en la segunda década de vida). Así mismo, el presente trabajo concuerda en que se presenta prevalencia de OI en pacientes que bordean la quinta década de vida en adelante ^7^^,^^9^^,^^13^^,^^28^, pero difieren de los estudios reportados por Zayet *et al*. ^37^, quienes reportan una mayor frecuencia en la tercera década de vida ^37^.

La importancia del hallazgo incidental de la OI ^31^^,^^34^ se evidencia cuando hay cambios posteriores en sus características morfométricas en los controles radiográficos, por lo cual se hace necesaria la exploración mediante tomografía computarizada de haz cónico. Esto se debe a las interacciones con los tratamientos realizados por las distintas especialidades odontoestomatológicas dadas a conocer en los reportes de casos publicados en la última década, que hacen referencia a situaciones clínicas como parestesia parcial del labio inferior o dificultad en el bloqueo regional del nervio mandibular ^39^^,^^40^, e incluso dolor orofacial con características de dolor neuropático por compresión de la OI al paquete vasculonervioso, lo que provoca neuropatías como neuropraxia, axonotmesis, neurotmesis ^33^^,^^41^. La OI puede causar desviación del eje axial de las piezas dentarias, apiñamiento, dificultades en el cierre de espacios e impactaciones dentarias ^5^^,^^6^^,^^19^^,^^34^^,^^42^^,^^43^. De la misma manera, puede causar la reabsorción externa del tercio apical de la raíz dentaria ^5^. 

La presencia de la OI modifica la planificación quirúrgica implantológica al requerir un protocolo específico de fresado con irrigación de solución isotónica a 5 °C ^22^. La remoción quirúrgica de la OI presenta una alta tasa de recidiva ^44^, por lo que un diagnóstico certero evitará biopsias innecesarias ^45^. Ghapanchi *et al*. ^46^demostraron una correlación estadísticamente significativa de la presencia de OI en pacientes con insuficiencia hepática crónica ^46^. Las evidencias reportadas denotan la importancia del diagnóstico diferencial de la OI y el seguimiento radiográfico de esta entidad clínica-radiológica.

## CONCLUSIÓN

La prevalencia de la OI en una población de la región Ayacucho fue del 17,4% con respecto al sexo, lo que evidencia una predilección por el sexo femenino en una proporción de 2 a 1, Con respecto a la edad, se evidencia que la OI se presenta con mayor frecuencia en la segunda década de vida.

## Fuente de financiamiento:

Autofinanciado
